# Image processing techniques for analyzing CT scan images towards the early detection of lung cancer

**DOI:** 10.6026/97320630015596

**Published:** 2019-09-12

**Authors:** Jeyaprakash Vasanth Wason, Ayyappan Nagarajan

**Affiliations:** 1 Department of Computer Applications, Alagappa University - Karaikudi, Tamilnadu, India

**Keywords:** CT lung cancer images, cancer detection, image processing

## Abstract

The application of image processing techniques for the analysis of CT scan images corresponding to lung cancer cells is gaining momentum in recent years. Therefore, it is of interest to discuss the use of a Computer-Aided Diagnosis (CAD) system using Computed Tomography (CT) images to help in the early diagnosis of lung cancer (to distinguish between benign and malignant tumors). We discuss and explore the design and significance of a CAD-CT image processed model in cancer diagnosis.

## Background

Small cell lung cancer and non-small cell lung cancer are common types of lung cancer [1]. The general symptoms of lung cancer include coughing up blood, chest pain, weight loss and loss of appetite, shortness of breath and feeling weak [2]. Early detection improves the survival rate from 15% to 50% [3]. However, there is a need to increase this survival rate more than the current value. Images generated by X-rays, Computed-Tomography (CT) scans, Magnetic Resonance Imaging (MRI) and others help in the early detection of lung cancer without surgery. The CT scan is the most recommended method which produces the 3D images of the lungs [3]. Mortality rate can be reduced by early detection and treatment of the disease. The process of early detection of cancer plays an important role to prevent cancer cells from multiplying and spreading. Existing lung cancer detection techniques are not adequate for providing accuracy. Hence, it is of importance to develop new methods for the early detection of lung cancer.

**Figure 1:**
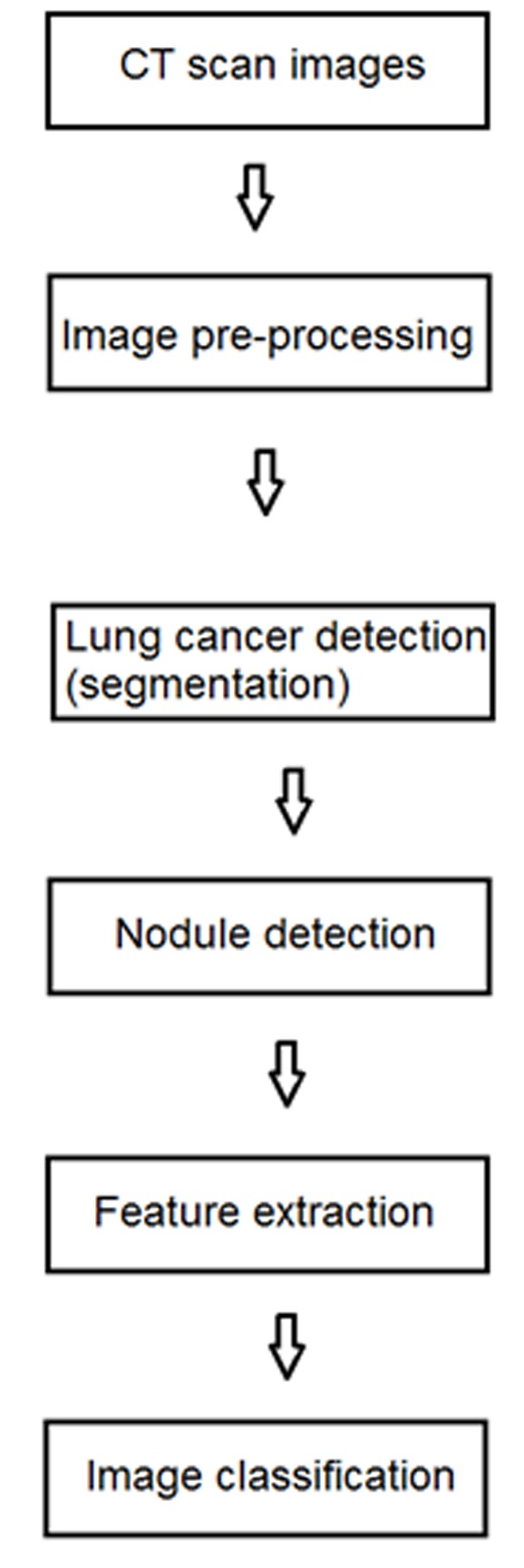
A flowchart describing the model design with features

The performance of Multilayer and Neural Network classifier trained by 11 training algorithms with Independent Component Analysis feature extraction is known [1]. A MATLAB based software tool to process the cancer image pre-processing is available [2]. It is shown that image processing techniques are very useful to detect tumor cells. A methodology based on average information parameters by utilizing image processing tools for lung cancer investigation is reported [4]. The authors revealed the real issue for the lung cancer diagnosis is the time constrictions for physical diagnosis. So they proposed a method which successfully rejected the null hypothesis test by implementing a standard statistical model. Dimililer et al. [5] used image pre-processing, image erosion, median filtering, thresholds and feature extraction for image processing techniques to apply on CT images. The authors discussed the development of an image processing algorithm for lung cancer detection using CT Images. A neuralnetwork- based system for the computer-aided detection of lung nodules in chest radiograms is shown [6]. They represent an artificial neural network-based lungs cancer detection system using CT images. An implementation and analysis of the image processing method for the detection of lung cancer is described [7]. The authors use color attribute in the feature extraction stage for the analysis of lung cancer using binarization to predict cancer in its earlier stage. Therefore, it is of concern to describe the use of a Computer-Aided Diagnosis (CAD) system using Computed Tomography (CT) images to help in the early diagnosis of lung cancer (to differentiate between benign and malignant tumors). We argue and search for the design and importance of a CAD-CT image processed model in cancer diagnosis as illustrated in Figure 1.

## Model Design

### Dataset:

A set of real patient CT scan images are obtained from the Lung Image Database Consortium (LIDC) archive is used in this analysis. LIDC database contains lung cancer screening CT images for development, training, and evaluation of computer-assisted diagnostic methods for lung cancer detection and diagnosis. The National Cancer Institute initiated it. It consists of 1018 cases of dataset contributed by seven academic centers and eight medical imaging companies [8]. Using Computed-Tomography (CT) images to ensure early diagnosis of lung cancer and differentiation between benign and malignant tumors [9] has developed computer-Aided Diagnosis (CAD) system. Computer-Aided Diagnosis (CAD) can be helpful for doctors to identify cancerous cells accurately [10].

### CAD system:

The CAD System has the following features: (1) It improves the diagnosis accuracy; (2) Assist in cancer detection at its earlier stage and (3) Reduces the time of the radiologist in evaluation.

## Discussion:

### Model description:

It is of interest to develop a CT scan image processed model for the early detection of lung cancer where (1) preprocessing using intensity measure helps to locate small particles in an image such as node, speculation and angular margin; (2) high detection and classification accuracy is established; and (3) removes noises that create false detection.

### The approach:

The first step is pre-processing of the image to locate particles using intensity measure. The processed image is segmented using a standard segmentation technique. Thus, cancer nodules are marked in the image. In addition to features like area, perimeter and eccentricity, other features like centroid, diameter and pixel mean intensity have been extracted during feature extraction. The classification module follows this where distinction between benign and malignant tumors based on CT scan images is established. Extracted features are used as training features and the corresponding trained model is generated for the classification followed by model evaluation for detection and classification with improved accuracy, specificity and sensitivity.

### Salient features:

The salient features of the method includes (1) image preprocessing by using filtering techniques and it smoothes the image and removes speckle noise; (2) It segments the cancer nodule from the CT scan image. The segmentation method separates and identifies the touching objects in the image. This feature helps in proper segmentation of cancer nodules when it is touching to other false nodules. (3) Features extraction where features like area, perimeter, centroid, diameter, eccentricity and mean intensity are extracted from the image. These features are used as training features to develop the classifier; and (4) The classification module classifies the detected nodule as malignant or benign by using the trained classification method.

### Strengths:

Strengths of the method includes: (1) The improved accuracy of cancer nodule detection; (2) classifies the detected lung cancer as malignant or benign; and (3) Removes the noises that create false detection of cancer.

## Conclusion

We describe and discuss the application of a Computer-Aided Diagnosis (CAD) system using Computed-Tomography (CT) images to help in the early diagnosis of lung cancer (to distinguish between benign and malignant tumors). We report a framework for the development of a model for early cancer detection using CADCT image analysis.
